# Reply to Yaffe, M.J. Correction for Self-Selection in Breast Cancer Screening. Comment on “Dibden et al. Worldwide Review and Meta-Analysis of Cohort Studies Measuring the Effect of Mammography Screening Programmes on Incidence-Based Breast Cancer Mortality. *Cancers* 2020, *12*, 976”

**DOI:** 10.3390/cancers15184577

**Published:** 2023-09-15

**Authors:** Amanda Dibden, Judith Offman, Stephen W. Duffy, Rhian Gabe

**Affiliations:** 1 Centre for Cancer Screening, Prevention and Early Diagnosis, Wolfson Institute of Population Health, Queen Mary University of London, Charterhouse Square, London EC1M 6BQ, UK; a.dibden@qmul.ac.uk (A.D.); j.m.offman@qmul.ac.uk (J.O.); 2 Centre for Evaluation and Methods, Wolfson Institute of Population Health, Queen Mary University of London, Charterhouse Square, London EC1M 6BQ, UK; r.gabe@qmul.ac.uk

In his Comment [1] on our review article “Worldwide Review and Meta-Analysis of Cohort Studies Measuring the Effect of Mammography Screening Programmes on Incidence-Based Breast Cancer Mortality” published in *Cancers* [2], Dr. Yaffe makes the point that, in combining results from multiple observational studies of cancer screening, using individual study-specific corrections for self-selection is preferable to using the same correction for all studies, since the factors affecting the bias will vary from setting to setting. We agree. Unfortunately, we did not obtain sufficient data from every study to calculate study-specific corrections, so as a second best, we applied a common independent estimate to all. In our paper, we stated that the corrected estimate of the relative risk showed significant heterogeneity, not the correction factor, as stated in the Comment, since we were unable to calculate the latter for every study.

Dr. Yaffe points out that there is little if any evidence for self-selection bias in the Canadian study of Coldman et al. [3]. This does appear to be the case, and it is likely that our common correction will over-correct the Canadian results. We suspect that there is agreement between us and Dr Yaffe that the correction is likely to be conservative overall. While it may lead to a slight underestimate of the benefit of participating in mammography screening, it does not lead to an overestimate.

## References

[1] Yaffe M.J. (2023). Correction for Self-Selection in Breast Cancer Screening. Comment on Dibden et al. Worldwide Review and Meta-Analysis of Cohort Studies Measuring the Effect of Mammography Screening Programmes on Incidence-Based Breast Cancer Mortality. *Cancers* 2020, *12*, 976. Cancers.

[2] Dibden A., Offman J., Duffy S.W., Gabe R. (2020). Worldwide Review and Meta-Analysis of Cohort Studies Measuring the Effect of Mammography Screening Programmes on Incidence-Based Breast Cancer Mortality. Cancers.

[3] Coldman A., Phillips N., Wilson C., Decker K., Chiarelli A.M., Brisson J., Zhang B., Payne J., Doyle G., Ahmad R. (2014). Pan-Canadian Study of Mammography Screening and Mortality from Breast Cancer. JNCI J. Natl. Cancer Inst..

